# Changes of immunogenic profiles between a single dose and one booster influenza vaccination in hemodialysis patients – an 18-week, open-label trial

**DOI:** 10.1038/srep20725

**Published:** 2016-02-12

**Authors:** Yu-Tzu Chang, Jen-Ren Wang, Meng-Te Lin, Chi-Jung Wu, Ming-Song Tsai, Chiang Lin Wen-Chi, Te-En Shih, Te-Hui Kuo, Eing-Ju Song, Junne-Ming Sung

**Affiliations:** 1 Institute of Clinical Medicine, College of Medicine, National Cheng Kung University, Tainan, Taiwan; 2 Department of Internal Medicine, National Cheng Kung University Hospital, College of Medicine, National Cheng Kung University, Tainan, Taiwan; 3 Department of Medical Laboratory Science and Biotechnology, College of Medicine, National Cheng Kung University, Tainan, Taiwan; 4 National Institute of Infectious Diseases and Vaccinology, National Health Research Institutes, Tainan, Taiwan; 5 Department of Internal Medicine, Kuo General Hospital, Tainan, Taiwan; 6 Department of Bioscience Technology, Chang Jung Christian University, Tainan, Taiwan

## Abstract

Annual influenza vaccination is recommended, but its efficacy in dialysis population is still controversial. Here we aimed to compare the dynamic changes of immune response between various influenza vaccination protocols in hemodialysis patients. A 18-week open label, non-randomized, controlled trial was conducted during 2011–2012. The efficacy between unvaccinated, one- and two-dose regimens were evaluated in 175 hemodialysis patients. Immunogenic profiles were assessed by hemagglutination-inhibition assays. At 3–9 weeks post-vaccination, antibody responses were similar between the one- and two-dose regimens, while the seroprotection rates (antibody titer ≥1:40) for influenza A were 55.6–82.5% in the adult (18–60 years) and 33.3–66.7% in the elderly (>60 years). Meanwhile, the seroprotection rates for influenza B were low (4.0–25.0%). By 18 weeks post-vaccination, the seroprotection rates for influenza A and B declined (0.0–33.3%) in both the adult and elderly receiving one- or two-dose regimens. Of dialysis patients, at most 2.4% developed moderate to severe adverse effects(myalgia and headache) after vaccination. In conclusion, the two-dose regimen could not improve immune responses than the one-dose regimen in hemodialysis patients; meanwhile the induced protective antibodies of both regimens could not be maintained for more than 4 months. Modification of current influenza vaccination strategy in dialysis population should be re-considered.

Through the mechanisms of antigenic shift and drift, the influenza virus has posed a persistent threat to humans and been responsible for several pandemics and numerous endemics over the past hundred years, including a devastating influenza pandemic in 1918^1^,^2^,^3^,^4^. Compared to the general population, dialysis patients are at 10- to 100-fold increased risk of mortality related to pneumonia and sepsis^5^. Thus, influenza prevention is a major focus in clinical practice for dialysis patients. To date, vaccination is still the primary strategy to prevent influenza infection although anti-viral agents are also of value^6^.

In the past two decades, the annual administration of one dose of trivalent influenza vaccine has been the standard care for dialysis patients. However, this suggestion is not based on solid evidence^7^. A growing body of evidence demonstrated there might exit significant biases while evaluating the influenza vaccine efficacy by observational studies. Among three studies investigating the effectiveness of influenza vaccine in dialysis population, inconsistent conclusions were made in spite of efforts to reduce possible confounding in their studies^8^,^9^,^10^. The possible explanations for the inconsistency might be related to estimation biases from various unmeasured confounders, such as healthy user effect^11^, the severity of the reference influenza season^7^, mismatch between vaccine-virus and circulating virus strains and the specificity of the clinical outcomes (causes of hospitalization, mortality and influenza-like illness). When evaluation the efficacy of influenza vaccination by serological outcomes, the results from these studies were also inconsistent^12^,^13^,^14^,^15^,^16^,^17^,^18^,^19^,^20^,^21^,^22^,^23^,^24^. Some studies affirmed the vaccine efficacy^12^,^14^,^15^,^17^,^18^,^19^,^21^,^24^ but some questioned its efficacy^13^,^16^,^20^,^22^,^23^. In addition, the lack of the consideration of baseline seroprotection level (SP_pre_)^13^,^17^, short follow-up period^14^,^15^,^16^,^18^,^19^,^20^,^23^, small sample size^17^,^18^,^19^, the addition of adjuvants in vaccines^12^,^20^,^24^ and analysis^12^,^17^,^19^,^24^ were not strictly followed by the age-specific criteria of European Union Committee For Proprietary Medicinal Products (CPMP)^25^. This results in the difficulty of comparing the results between these studies and interpretations of these studies should be cautious.

To improve the efficacy of influenza vaccination in dialysis patients, the strategy of one booster dose has been applied. Although most of the studies indicated one booster dose could not enhance the immune response^12^,^17^,^20^,^21^,^22^,^24^, the analyses were universally based on the titer levels at one month after the booster dose. Because of the defective immune system of dialysis patients, the duration of induced antibody existence after vaccination has not been evaluated either in the one dose or one booster dose regimen. Whether one booster dose can maintain longer duration of protective antibody level than one standard dose is still unknown. In this study, we aimed to compare the efficacy, safety and the differential changes of serial antibody responses between the unvaccinated, one-dose and two-dose regimens (3 weeks apart between vaccinations) of the non-adjuvanted trivalent influenza vaccine throughout an 18-week follow-up period in patients undergoing dialysis. The setting of an 18-week follow-up guaranteed us to evaluate whether the vaccine-induced antibody levels could be maintained at least till the end of the influenza season. The analysis would be performed by age stratification (≤60 or >60) because of the different evaluation criteria of vaccine efficacy suggested by the European Union CPMP.

## Methods

### Study design and enrolled subjects

During the 2011–2012 influenza season, we conducted this open-label, controlled trial to evaluate the efficacy and safety of the trivalent seasonal influenza vaccine for hemodialysis patients. Four dialysis centers (Kuo General Hospital, Yan-Ta Shiang Clinic, Yi-Lin Clinic, Chong-Ren Medical Clinic) participated in the study. The Institutional Review Board of National Cheng Kung University Hospital (IRB number: BR-100-0086) and Kuo General Hospital (IRB number: ER-11-K008) approved this study, which was registered in Clinicaltrials.gov (NCT01512056), and the methods were carried out in accordance with the approved current guidelines. Males or non-pregnant females aged ≥18 years and receiving hemodialysis for ≥3 months were eligible for enrollment into the study. Key exclusion criteria included receiving the 2010–2011 influenza vaccine within a 6-month interval, egg hypersensitivity, a personal or family history of Guillain-Barré Syndrome, fever within 1 week or influenza-like illness within 3 days prior to vaccination, human immunodeficiency virus infection or taking any immunosuppressive agent for 3 subsequent months before enrollment, receiving any blood products or having been hospitalized for any illness within the past 3 months.

**Figure 1:**
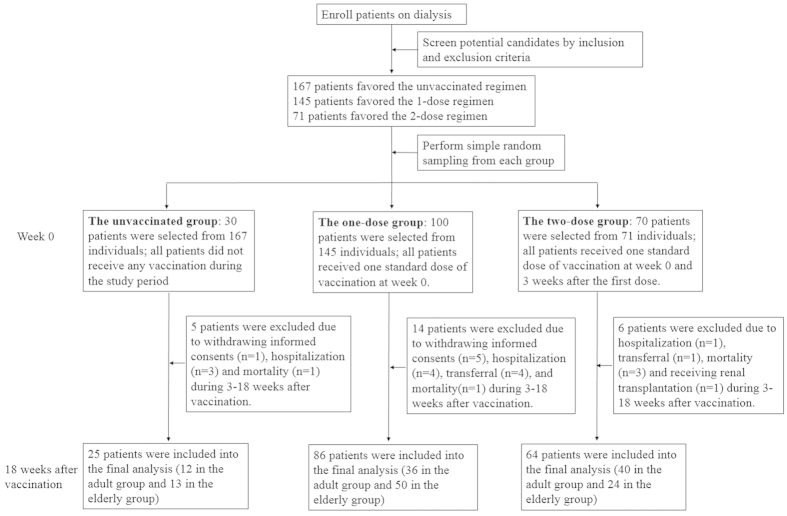
The flow chart and the immunization protocol of the study.

In this study, individuals were assigned to any group according to their own free will. The participants who refused to receive the vaccination (the unvaccinated group) were served as the negative control group to monitor local influenza virus activity throughout the study period. The one-dose group received one standard dose when enrolled. Those in the two-dose group received the second vaccination 3 weeks after the first dose (Fig. 1). Instead of power calculation, the sample size in each intervention group was at least 50, which was recommended by European guidelines for influenza trials^26^.

At the initial screening, 167, 145 and 71 patients were eligible to join in the unvacinnated, one-dose and two-dose vaccination groups, respectively (Fig. 1). To maximize patient number in the two-dose group, concerning limited manpower and reducing selection bias by physicians, we performed simple random sampling to choose 30 of the 167, 100 of the 145 and 70 of the 71 patients from these three vaccination groups. No statistically significant differences in age and gender distribution were noted after sampling (all *p* values within 0.85–0.98). Serum samples were collected at week 0 (before the vaccination), and at 3, 6, 9 and 18 weeks after the entering of the study (the unvaccinated group) or after the first vaccination (the one- and two-dose group). Informed written consent was obtained from all study participants.

It is well known that dialysis patients have impaired innate and adaptive immunity and they are prone to have poor immune response elicited by vaccination. When evaluating the vaccine efficacy by HI titers in dialysis population, it would be hard to tell which factor is the major determinant for the suboptimal or poor immune response after vaccination once observed: the poor immunogenicity of vaccine itself or the impaired immune system of dialysis patients. Therefore, another clinical trial performed in normal individuals (n = 114) by using the same formula of influenza vaccine was set as an active control group (ClinicalTrials.gov identifier number: NCT01356316) for comparison.

### The protocol and safety monitoring of influenza vaccination

The single dose of the trivalent, non-adjuvanted and inactivated split-viron vaccine (AdimFlu-S), manufactured by Adimmune Corporation (Taichung, Taiwan), contained 15 μg of each viral hemagglutinin (HA) antigen, including A/California/7/2009 A(H1N1)pdm09 (Reassortant NYMC X-181), A/Perth/16/2009 (H3N2) (Reassortant NYMC X-187) and B/Brisbane/60/2008, as recommended by the World Health Organization (WHO) for the 2011–2012 influenza season in the Northern Hemisphere. For each vaccination, the participants received one dose of vaccine by intramuscular injection into the deltoid region. Adverse events of all subjects were monitored during the study period. Each subject was instructed to record systemic and local adverse effects once daily on a diary card for 7 days after vaccination.

### Immunogenicity established by hemagglutination-inhibition (HI) assays and clinical events related to the vaccination

All obtained serum samples were stored at −80 °C after sampling until analysis of HI assays. The samples were re-labeled with serial numbers according to the randomization list for serum samples before being sent to the analysis laboratory. The HI assays were measured according to the standard protocol suggested by the WHO^27^, and were used to assess the immune responses based on the international guidelines to evaluate the efficacy of influenza vaccines^25^,^28^. The HI titers were recorded as the reciprocal of the highest serum dilution test that completely inhibited hemagglutination. Antibody titers less than 1:10 were recorded as 1:5 in the statistical analysis. All measurements were performed on duplicate samples. Four immunogenicity parameters were used to evaluate the efficacy of the influenza vaccine: (a)seroprotection rate (HI antibody titer ≥ 1:40); (b)seroconversion rate (≥4-fold HI titer with the titer ≥1:40 after vaccination); (c)seroresponse rate (≥4-fold increase in the HI antibody titer after vaccination); (d)geometric mean titer (GMT) and the fold of rise in GMT. In addition, information related to all-cause mortality and hospitalization of the study subjects would be ascertained by both direct telephone contact and medical records of each dialysis center during the study period.

### Statistical analysis

Differences between groups were compared using one-way analysis of variance for continuous variables, and the chi-square test or Fisher’s exact test for categorical variables. The Wilcoxon rank sums test was used to test differences of continuous variables between the two groups if a non-normal distribution was noted. The Clopper-Pearson method was used to estimate the 95% confidence intervals (CIs) of seroprotection, seroresponse and seroconversion rates. GMTs and the corresponding 95% CIs were calculated by taking the exponential of the log of the means and their 95% CIs. Differences in pre-vaccination GMT and fold increase of GMT between two groups were compared by the independent *t*-test after log transformation. The difference-in-difference method was used to compare differences in *log*-transformed GMTs between two groups after taking the effect of *log*-transformed GMTs before vaccination into consideration. To identify differences in seroprotection and seroresponse rates defined by repeated measurements of HI titers between different vaccination groups, the logistic regression models with generalized estimation equations (GEE) based on exchangeable working correlation matrixes were constructed and adjusted for possible risk factors of immune response to influenza vaccination in dialysis population^12^,^23^,^24^. A two-side *p*-value less than 0.05 was considered to be statistically significant. All statistical analyses were performed with SAS version 9.4 (SAS Institute, Cary, NC). Graphs were constructed by GraphPad Prism version 5.01 (GraphPad Software, Inc., La Jolla, CA).

## Results

### Baseline characteristics of the participants

During the 18-week study period, 25 patients were excluded due to withdrawing informed consent (n = 6), being hospitalized (n = 8), transferring to the other dialysis centers (n = 5), mortality (n = 5) and receiving a kidney transplantation (n = 1). Only one patient in each of the single- and two-dose groups was admitted due to pneumonia. No cases of mortality were attributable to pneumonia or related complications. Therefore, only 25, 86 and 64 dialysis patients in the unvaccinated, one-dose and two-dose vaccination groups, respectively, completed the five-time point serum collection throughout the study (Fig. 1). There were no significant differences in age, gender or underlying diseases between the three groups (Table 1). Kt/V (K: dialyzer clearance of urea, t: duration of dialysis, V: volume of distribution of urea), a parameter used to evaluate dialysis adequacy, was significantly different between the three groups (p = 0.017), and the lowest level (1.45 ± 0.28) was noted in the two-dose vaccination group. However, this lowest value was still higher than the suggested level for dialysis adequacy (Kt/V ≥1.2, defined by the KDOQI guideline^29^). In contrast, handgrip strength, a surrogate marker of nutrition^30^, was highest in the two-vaccination group. The self-reported number of influenza vaccinations in the previous 2 years varied, and the participants in the two-vaccination group had the highest rate of vaccinations in these two years.

### Dynamic change of immunogenicity based on HI assays and clinical outcomes in the different vaccination groups

**Figure 2:**
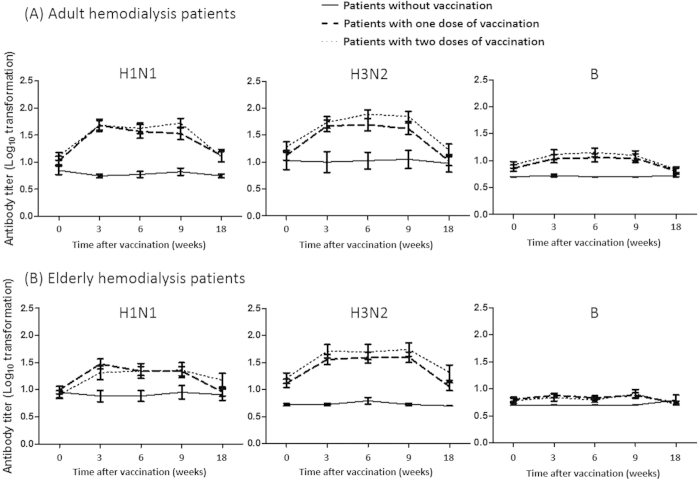
The dynamic changes of log_10_ transformed mean level and the associated 95% confidence interval of anti-hemagglutinin antibody titers in (**a**) adult and (**b**) elderly hemodialysis patients of the unvaccinated-, one- and two-dose of seasonal trivalent influenza vaccine during the whole study period (18 weeks).

In the unvaccianted group, no significant dynamic changes in the various parameters of HI titers were observed in either the adult (18–60 years)(Table 2 and Fig. 2) or elderly (>60 years)(Table 3 and Fig. 2) patients throughout the study period.

In the adult patients, there were no significant differences in baseline seroprotection rates and GMTs against all three virus strains between the one-dose and the two-dose vaccination groups (Table 2 and Fig. 2). During 3 to 9 weeks after vaccination, a single dose of vaccination elicited a statistically significant immune response compared with the unvaccinated group. Most values of the parameters of fold increases of GMTs and seroresponse rates for H1N1 and H3N2 were slightly higher than 2.5 and 40%, which were the efficacy thresholds suggested by the CPMP^25^ and the Food and Drug Administration in the United States^28^. However, the seroprotection rates did not reach more than 70%. A booster vaccination elicited higher immunogenicity profiles than in the one-vaccination group during 6 to 9 weeks after the first vaccination, although without statistical significant difference. Eighteen weeks after the first vaccination, dramatic decreases of all immunogenicity profiles were observed in both the one- and two-dose vaccination groups. The immune response against influenza B was poor throughout the study period. In the elderly patients, similar results related to the dynamic changes of all immunogenic profiles during the study period were observed as in the adult patients (Table 3 and Fig. 2).

When considering the effect of vaccination on all-cause mortality and hospitalization for pneumonia during the follow-up period by intention-to-treat analysis (n = 200), there were no significant differences of mortality or pneumonia rates between the three groups (*P* value = 0.356 and 1.000, respectively).

### Comparison of HI immunogenicity between healthy individuals and dialysis patients using the same formula of influenza vaccine

When comparing HI-based immunogenic profiles between the healthy individuals and dialysis patients, no statistically significant differences in baseline seroprotection rates and GMTs were noted between these two groups (Table 4). After one dose of vaccination, many of the parameters of HI immunogenicity were significantly higher in the healthy individuals than in the dialysis patients for all three vaccine strains. Similarly, the immune response for influenza B strain was still weaker than those for the influenza A strains.

### Evaluation of the effects of different vaccination protocols using a multivariate logistic regression model with generalized estimating equations (GEE)

To compare whether the administration of two-dose vaccinations induced a higher possibility of achieving seroprotection or seroresponse than one-dose vaccination throughout the whole study period, we used a logistic regression model with GEE by taking into account the statistical intercorrelation between the repeated four time-point of seroprotection and seroresponse measurements from the same individual after the first vaccination (Table 5). After adjusting for possible risk factors of immune response to influenza vaccination in dialysis population^12^,^23^,^24^, the two-dose regimen did not induce a higher chance of seroprotection or seroresponse than the one-dose regimen for all three virus strains. The presence of baseline SP_pre_ was the most important determinant to predict the possibility of seroprotection or seroresponse after vaccination.

Furthermore, we also constructed GEE models to investigate the time effect on the secular change of HI titers in different vaccination groups. Since interaction of time and the various vaccination groups for log_10_-transformed HI titers was noted, we analyzed the results under stratification by various vaccination regimens. Similar to the results revealed in Fig. 2, log_10_-transformed HI titers at 18 weeks after vaccination would drop to similar level as those in baseline period in either one- or two-dose group (Supplementary Table 1).

### Adverse events after vaccination in the dialysis patients

Overall, most of the systemic or local adverse effects were mild in the dialysis patients receiving either the first or second vaccination (Table 6). Only up to 2.4% of the participants developed moderate to severe adverse effects (muscle aches and headache) within 1 week after vaccination.

## Discussion

In this study, our results revealed that administration of one booster dose of a non-adjuvanted, trivalent inactivated influenza vaccine at 3 weeks after the first dose could neither result in a significantly additional improvement in any immunogenicity profile nor reduce the all-cause mortality or hospitalization for pneumonia rates than the one-dose regimen. Furthermore, the induced protective antibodies level could not be sustained for more than 18 weeks in either the one- or two-dose vaccination regimens. This is in contrast to the findings observed in normal adult and elderly populations^31^,^32^,^33^, and highlight the protective effect of both regimens could not be even maintained throughout the whole influenza season. Therefore, modification of the current influenza vaccination strategy in dialysis patients, such as the supplement of one booster dose at the middle of the influenza season, might be necessary to provide long enough protection for reducing influenza infection.

Several studies have explored the effect of one booster vaccination in dialysis patients^12^,^17^,^20^,^21^,^22^,^24^, and only one study reported positive results of a dose-dependent immune response induced by influenza vaccination^24^. One plausible biological mechanism may be that the administration of a specific amount of influenza virus antigen only elicited a fixed immune response, and that repeated exposure to the same amount of antigen within a relatively short period may not further enhance the immune response. This might explain why dialysis patients could have at least sub-optimal response to the first dose of vaccination but not to the booster dose in our study. Further studies are needed to clarify at which time point repeated vaccinations would be beneficial for dialysis patients.

In our study, we set the unvaccinated group as a negative control group and another clinical trial performed in healthy individuals as an active control group. When assessing the dynamic change of HI titers during the study period, intercurrent influenza infection, either symptomatic or asymptomatic, can induce the increment of HI titers and is a possible bias for interpretation of vaccination effects. The setting of the unvaccinated group could help us to monitor if there is any spreading of influenza infection within our study population throughout the study period. This is because both patients with or without vaccination stay in closed environment during their dialysis process, which might potentially facilitate the influenza transmission, and any elevation of HI titers in the unvaccinated group can indicate the spreading of influenza infection in our study population. Since no obvious elevation of various serological profiles could be observed during the study period in the unvaccinated group, we could tentatively conclude that the increased HI titers in the vaccinated group were mainly related to the effect of vaccination. Besides, the suboptimal immune response after vaccination could only be observed in dialysis patients but not in healthy subjects (Table 4). Therefore, the suboptimal immune response could be attributable to the impaired immune system of dialysis patients rather than the immunogenicity of the vaccine.

Although our results showed only sub-optimal antibody responses induced by the non-adjuvanted influenza vaccination, annual influenza vaccinations are still suggested because they may provide extra clinical benefits for dialysis patients. Hemodialysis patients are at a high risk of respiratory tract infections, and the stay in closed environments during dialysis process may foster transmission of influenza. Considering the effect of herd immunity, the administration of a influenza vaccine could reduce the spread of influenza to unvaccinated patients and serve as a firewall in dialysis centers. In addition, repeated exposure to the same vaccine virus strain in the previous consecutive years may increase the probability of achieving SP_pre_ the following year^12^,^34^,^35^. Our results also showed that baseline SP_pre_ was a major predictor of seroprotection after vaccination (Table 5). Therefore, annual vaccinations should still be encouraged in clinical practice.

Recently, Bond *et al.* demonstrated seasonal variations in the protective effect of influenza vaccines in dialysis patients, with the protective effect against mortality attenuating gradually over time^9^. They speculated that this may be due to some unvaccinated patients dying early leaving more healthy unvaccinated patients in later analysis. In addition, the decline of protective antibodies over time as found in our study might be an alternative explanation for this finding. This also suggests the need for differential management strategies targeting the maintenance of adequate antibody levels at time periods after the vaccination.

In our study, it is interesting to find out the induced antibody production against the influenza B type is consistently lower than those of the influenza A types (H1N1, H3N2). Among the studies evaluating the efficacy of trivalent influenza vaccines based on HI assays in dialysis population^12^,^14^,^15^,^16^,^17^,^18^,^19^,^20^,^21^,^22^, we did not find any specific virus type had consistently superior or inferior immunogenicity than the others. The results evaluated by seroresponse, seroconversion or fold of increased HI titers were diverse between different types in different studies. In addition, the low protective antibody production against influenza B type were observed both in dialysis patients and health individuals. Therefore, the most possible explanation for this phenomenon would be the low immunogenicity of the selected influenza B component in the vaccine we used in this study.

Dialysis patients are found to have defective immune system and are characterized by dull immune response to vaccination. Uremic toxins are speculated to be responsible for this phenomenon and it is suggested that dialysis therapy and increment of dialysis adequacy (Kt/V) can optimize immune function^36^,^37^. However, limited information exits concerning the effect of dialysis adequacy on vaccine-induced immunity. As suggested by Kovacic *et al*, higher Kt/V values were associated with better HBV vaccine reaction^38^. Nevertheless, no association between Kt/V and antibody response was found in the influenza vaccine trials^12^,^23^,^24^. Analysis by either univariate or multivariate GEE models in our study also showed no significant association (data not shown, *p* values in a range of 0.107 to 0.964). Altogether, we suggested the effect of dialysis adequacy on immune responses elicited by influenza vaccine is minimal.

There are several limitations to the current study. First, this was not a randomized controlled trial, and unmeasured confounders may have biased the estimation of the efficacy of the influenza vaccine. However, we included a negative control group, two active control groups and repeated measurements of various immunogenic profiles within an 18-week period. Several potential confounders were also adjusted for in the GEE models to reduce bias as far as possible. Besides, handgrip strength and previous vaccination episodes, the surrogate parameters of nutrition and health behavior, respectively, were better in the two-dose group than the one-dose group. Therefore, the impaired health status might not be the reason to explain why the two-dose regimen could not induce higher protective antibody titers than the one-dose regimen. Second, patients with certain specific comorbidities and those in an unstable condition were excluded before enrollment. The extrapolation of our study results should be made with caution. However, the inclusion of these patients into the study may have only resulted in an overall reduced vaccine efficacy. Third, we stratified our study population by age in order to follow the CPMP guidelines for the evaluation of vaccine efficacy^25^. This led to a small number of patients in some of the stratifications, and the results derived from these stratifications with limited sample size should be interpreted cautiously. Fourth, since there is only a small difference of various serological parameters (effect size) between the study groups, our study might be underpowered to correctly detect such a small difference between the one- and two-dose regimens. Accordingly, it might be too premature to conclude that the administration of one booster dose does not make a difference in serological outcomes. Finally, the vaccine efficacy in this study was only evaluated by HI assays and we should keep in mind that the change of serum HI titers is not always consistent with the alteration of clinical outcomes.

In conclusion, the application of two-dose regimen of non-adjuvanted, trivalent inactivated influenza vaccine could not induce significantly higher protective antibodies or reduce all-cause mortality/hospitalization rates than the one-dose regimen. Furthermore, the decline in protective antibodies 3 to 4 months after the vaccination suggests the need for differential vaccination programs for dialysis patients, especially when only non-adjuvanted influenza vaccines are available. Further studies are needed to clarify whether the use of adjuvanted vaccines or vaccines containing higher antigen dosages will lead to higher antibody levels for a longer duration than non-adjuvanted or standard dosage vaccines in dialysis patients.

## Additional Information

**How to cite this article**: Chang, Y.-T. *et al.* Changes of immunogenic profiles between a single dose and one booster influenza vaccination in hemodialysis patients- an 18-week, open-label trial. *Sci. Rep.* **6**, 20725; doi: 10.1038/srep20725 (2016).

## Supplementary Material

Supplementary Information

## Figures and Tables

**Table 1:** The baseline characteristics of the enrolled participants between the groups of the unvaccinated, one- or two-dose of influenza vaccination.

	All (n = 175)	The unvaccinated group (n = 25)	The one-dose vaccination group (n = 86)	The two-dose vaccination group (n = 64)	P value^a^
Age (years)	59.85 ± 12.92	60.24 ± 14.07	61.44 ± 13.28	57.56 ± 11.79	0.190
Men/Women (n)	96/79	11/14	46/40	39/25	0.331
Dry weight (Kgw)	59.74 ± 14.45	55.69 ± 11.91	59.43 ± 14.24	61.68 ± 15.42	0.215
Underlying disease					
Hypertension (n, %)	112 (64%)	17 (68%)	57 (66.3%)	38 (59.4%)	0.618
Diabetes (n, %)	67 (38.3%)	9 (36%)	35 (40.7%)	23 (35.9%)	0.812
Hepatitis B (n, %)	28 (16%)	4 (16%)	12 (14%)	12 (18.8%)	0.719
Hepatitis C (n, %)	28 (16%)	3 (12%)	18 (20.9%)	7 (10.9%)	0.261
Cardiovascular disease (n, %)	49 (28%)	5 (20%)	29 (33.7%)	15 (23.4%)	0.240
Hematology					
White blood cell (10^3^/μL)	6.73 ± 2.14	6.55 ± 2.08	6.91 ± 2.26	6.57 ± 2.03	0.561
Hematocrit (%)	32.25 ± 5.11	30.82 ± 5.61	32.30 ± 5.45	32.71 ± 4.38	0.301
Platelet (10^3^/μL)	199.39 ± 69.23	206.79 ± 60.76	201.27 ± 73.49	194.14 ± 67.03	0.706
Biochemistry					
BUN (mg/dL)	65.86 ± 18.48	74.25 ± 22.71	67.25 ± 16.80	60.90 ± 17.68	0.006
Creatinine (mg/dL)	10.11 ± 2.46	9.84 ± 2.52	9.93 ± 2.30	10.44 ± 2.65	0.396
Glucose AC (mg/dL)	124.73 ± 69.18	124.54 ± 55.94	127.77 ± 64.55	120.81 ± 79.56	0.834
Calcium (mg/dL)	9.43 ± 0.74	9.23 ± 0.93	9.48 ± 2.30	9.45 ± 0.60	0.330
Phosphate (mg/dL)	4.99 ± 1.47	5.04 ± 1.29	5.23 ± 1.63	4.67 ± 1.26	0.070
Sodium (mmol/L)	135.97 ± 9.90	136.36 ± 3.08	135.56 ± 13.81	136.36 ± 3.33	0.871
Potassium (mmol/L)	4.63 ± 0.77	4.73 ± 0.82	4.66 ± 0.80	4.55 ± 0.71	0.523
Albumin (mg/dL)	3.98 ± 0.38	4.01 ± 0.33	3.95 ± 0.40	4.02 ± 0.37	0.479
Total-cholesterol (mg/dL)	173.02 ± 37.13	172.33 ± 39.78	172.46 ± 36.73	174.02 ± 37.23	0.965
Triglyceride (md/dL)	128.09 ± 76.95	132.67 ± 83.37	139.35 ± 77.22	111.59 ± 7.27	0.089
iPTH (pg/ml)	290.90 ± 354.55	201.60 ± 211.14	342.32 ± 446.12	256.10 ± 231.78	0.140
Ferritin (ng/ml)	413.07 ± 388.97	575.39 ± 423.63	368.51 ± 325.09	411.37 ± 440.35	0.070
Dialysis adequacy					
Single pool Kt/V	1.51 ± 0.30	1.65 ± 0.33	1.52 ± 0.30	1.45 ± 0.28	0.017
Urea reduction rate (%)	72.58 ± 8.74	74.88 ± 7.33	72.73 ± 9.03	71.50 ± 8.77	0.266
Anthropometric markers					
Waist circumference (cm)	85.58 ± 12.12	84.63 ± 11.16	85.56 ± 11.78	85.93 ± 13.01	0.911
Hip circumference (cm)	90.51 ± 9.22	91.20 ± 9.12	90.94 ± 9.71	89.71 ± 8.68	0.691
Waist-hip ratio	0.94 ± 0.09	0.93 ± 0.06	0.94 ± 0.06	0.96 ± 0.12	0.290
Body mass index	22.65 ± 4.23	21.23 ± 4.05	22.62 ± 4.09	23.21 ± 4.40	0.147
Body adiposity index	26.06 ± 4.91	26.65 ± 4.89	26.35 ± 5.10	25.49 ± 4.70	0.498
Handgrip strength (Kgw)	18.55 ± 11.35	16.50 ± 9.07	16.95 ± 11.31	21.57 ± 11.76	0.031
Previous vaccination history					
during 2009-2010	110 (62.9%)	11 (44.0%)	48 (55.8%)	51 (79.7%)	0.001
during 2010-2011	109 (62.3%)	4 (16.0%)	57 (66.3%)	48 (75.0%)	<0.001

**Table 2:** Dynamic changes of hemagglutination-inhibition (HI) immunogenicity during the 18-week study period in adult dialysis patients (age between 18–60 years-old).

Dialysis population									
Immunogenicity end point	The unvaccinated group (n = 12)			The one-dose vaccination group (n = 36)			The two-dose vaccination group (n = 40)		
	H1N1	H3N2	B	H1N1	H3N2	B	H1N1	H3N2	B
Baseline									
Seroprotection rate % (95% CI)	8.3% (0.2–38.5)	25.0% (5.5–57.2)	0.0% (0–26.5)	22.2% (10.1–39.2)	27.8% (14.2–45.2)	8.3% (1.8–22.5)	22.5% (10.8–38.5)	32.5% (18.6–49.1)	10.0% (2.8–23.7)
Geometric mean titer (95% CI)	7.1 (4.7–10.5)	10.6 (4.5–24.8)	5.0 (5.0–5.0)	11.4 (7.8–16.8)	12.8 (8.5–19.3)	7.3 (5.6–9.7)	13.2 (9.4–18.5)^a^	17.7 (11.4–27.4)	8.1 (6.2–10.7)^a^
3 weeks later									
Seroprotection rate % (95% CI)	0.0% (0–26.5)	16.7% (2.1–48.4)	0.0% (0–26.5)	66.7% (49.0–81.4)	69.4% (51.9–83.7)^a^	22.2% (10.1–39.2)	67.5% (50.9–81.4)	67.5% (50.9–81.4)^a^	25.0% (12.7–41.2)
Geometric mean titer (95% CI)	5.6 (4.7–6.7)	10.0 (3.8–26.5)	5.3 (4.7–6.0)	47.6 (28.9–78.4)^a^	44.0 (26.5–73.2)^a^	10.6 (7.5–15.1)	49.3 (32.1–75.7)^a^	59.6 (36.4–97.7)^a^	13.0 (8.6–19.5)
Fold increase of GM titer (95% CI)	0.8 (0.5–1.2)	0.9 (0.6–1.5)	1.1 (0.9–1.2)	4.2 (2.4–7.3)^a^	3.4 (2.0–5.9)^a^	1.4 (1.1–2.0)	3.7 (2.4–5.7)^a^	3.4 (2.0–5.6)^a^	1.6 (1.0–2.4)
Seroresponse rate % (95% CI)	0.0% (0–26.5)	0.0% (0–26.5)	0.0% (0–26.5)	52.8% (35.5–69.6)^a^	38.9% (23.1–56.5)^a^	19.4% (8.2–36.0)	47.5% (31.5–63.9)^a^	40.0% (24.9–56.7)^a^	27.5% (14.6–43.9)^a^
Seroconversion rate % (95% CI)	0.0% (0–26.5)	0.0% (0–26.5)	0.0% (0–26.5)	44.4% (27.9–61.9)^a^	38.9% (23.1–56.5)^a^	13.9% (4.7–29.5)	40.0% (24.9–56.7)^a^	30.0% (16.6–46.5)^a^	17.5% (7.3–32.8)
6 weeks later									
Seroprotection rate % (95% CI)	0.0% (0–26.5)	16.7% (2.1–48.4)	0.0% (0–26.5)	58.3% (40.8–74.5)	63.9% (46.2–79.2)^a^	22.2% (10.1–39.2)	62.5% (45.8–77.3)	82.5% (67.2–92.7)^a^	25.0% (12.7–41.2)
Geometric mean titer (95% CI)	5.9 (4.5–7.8)	10.6 (4.8–23.2)	5.0 (5.0–5.0)	37.0 (21.0–65.2)^a^	45.8 (27.7–75.6)^a^	10.8 (7.7–15.3)	43.6 (27.6–69.0)^a^	81.4 (55.4–119.5)^a^	13.7 (9.6–19.5)^a^
Fold increase of GM titer(95% CI)	0.8 (0.7–1.0)	1.0 (0.6–1.7)	1.0 (1.0–1.0)	3.2 (1.8–5.8)^a^	3.6 (2.1–5.9)^a^	1.5 (1.1–2.0)	3.3 (2.0–5.3)^a^	4.6 (2.9–7.3)^a^	1.7 (1.2–2.4)
Seroresponse rate % (95% CI)	0.0% (0–26.5)	8.3% (0.2–38.5)	0.0% (0–26.5)	41.7% (25.5–59.2)^a^	41.7% (25.5–59.2)^a^	22.2% (10.1–39.2)	50.0% (33.8–66.2)^a^	55.5% (38.5–70.7)^a^	20.0% (9.1–35.7)
Seroconversion rate % (95% CI)	0.0% (0–26.5)	0.0% (0–26.5)	0.0% (0–26.5)	36.1% (20.8–53.8)^a^	36.1% (20.8–53.8)^a^	13.9% (4.7–29.5)	37.5% (22.7–54.2)^a^	42.5% (27.0–59.1)^a^	12.5% (4.2–26.8)
9 weeks later									
Seroprotection rate % (95% CI)	0.0% (0–26.5)	25.0% (5.5–57.2)	0.0% (0–26.5)	55.6% (38.1–72.1)	63.9% (46.2–79.2)^a^	25.0% (12.1–42.2)	67.5% (50.9–81.4)	75.0% (58.8–87.3)^a^	22.5% (12.7–41.2)
Geometric mean titer (95% CI)	6.7 (4.7–9.5)	11.2 (4.8–26.5)	5.0 (5.0–5.0)	33.6 (20.2–56.0)^a^	39.2 (24.0–64.3)^a^	11.2 (7.9–16.0)^a^	53.7 (35.4–81.5)^a^	73.4 (47.6–113.1)^a^	11.9 (8.0–17.6)
Fold increase of GM titer (95% CI)	0.9 (0.8–1.2)	1.1 (0.8–1.4)	1.0 (1.0–1.0)	2.9 (1.8–4.9)^a^	3.1 (1.8–5.1)^a^	1.5 (1.1–2.1)	4.1 (2.8–5.9)^a^	4.1 (2.5–6.8)^a^	1.5 (1.0–2.3)
Seroresponse rate % (95% CI)	0.0% (0–26.5)	8.3% (0.2–38.5)	0.0% (0v26.5)	38.9% (23.1–56.5)^a^	41.7% (25.5–59.2)^a^	19.4% (8.2–36.0)	57.5% (40.9–73.0)^a^	55.5% (38.5–70.7)^a^	22.5% (10.8–38.5)
Seroconversion rate % (95% CI)	0.0% (0–26.5)	0.0% (0–26.5)	0.0% (0–26.5)	33.3% (18.6–51.0)^a^	36.1% (20.8–53.8)^a^	13.9% (4.7–29.5)	50.0% (33.8–66.2)^a^	45.0% (29.3–61.5)^a^	15.0% (5.7–29.8)
18 weeks later									
Seroprotection rate % (95% CI)	0.0% (0–26.5)	16.7% (2.1–48.4)	0.0% (0–26.5)	25.0% (12.1–42.2)	22.2% (10.1–39.2)	5.6% (0.7–18.7)	25.0% (12.7–41.2)	32.5% (18.6–49.1)	10.0% (2.8–23.7)
Geometric mean titer (95% CI)	5.6 (4.7–6.7)	9.4 (4.1–21.6)	5.3 (4.7–6.0)	12.6 (7.6–20.9)	10.0 (6.8–14.6)	6.3 (5.0–8.0)	13.7 (8.5–21.9)	17.7 (10.3–30.3)	6.8 (5.3–8.7)
Fold increase of GM titer (95% CI)	0.8 (0.5–1.2)	0.9 (0.5–1.5)	1.1 (0.9–1.2)	1.1 (0.6–2.1)	0.8 (0.5–1.3)	0.9 (0.7–1.1)	1.0 (0.6–1.9)	1.0 (0.5–1.9)	0.8 (0.6–1.2)
Seroresponse rate % (95% CI)	0.0% (0–26.5)	8.3% (0.2–38.5)	0.0% (0–26.5)	22.2% (10.1–39.2)	19.4% (8.2–36.0)	2.8% (0.1–14.5)	30.0% (16.6–46.5)^a^	25.0% (12.7–41.2)	10.0% (2.8–23.7)
Seroconversion rate % (95% CI)	0.0% (0–26.5)	0.0% (0–26.5)	0.0% (0–26.5)	19.4% (8.2–36.0)	13.9% (4.7–29.5)	0.0% (0.0–9.7)	20.0% (9.1–35.7)	17.5% (7.3–32.8)	5.0% (0.6–16.9)

Abbreviations: CI: confidence interval; GM: geographic mean; Definition of seroprotection: HI titers ≥ 1:40; Seroresponse: ≥ 4-fold increase in antibody titer after vaccination; Seroconversion: ≥ 4-fold or more increase in HI titer and HI titer ≥ 1:40 after vaccination.

^a^P value < 0.05 compared with the unvaccinated group.

^b^There were no statistical differences between all immunogenic profiles between the one-dose and two-dose vaccination groups.

**Table 3:** Dynamic changes of hemagglutination-inhibition (HI) immunogenicity during the 18-week study period in the elderly dialysis patients (age over 60 years-old).

Dialysis population									
Immunogenicity end point	The unvaccinated group (n = 13)			The one-dose vaccination group (n = 50)			The two-dose vaccination group (n = 24)		
	H1N1	H3N2	B	H1N1	H3N2	B	H1N1	H3N2	B
Baseline									
Seroprotection rate % (95% CI)	7.7% (0.2–36.0)	0.0% (0–24.7)	0.0% (0–24.7)	16.0% (7.2–29.1)	32.0% (19.5–46.7)^a^	8.0% (2.2–19.2)	4.2% (0.1–21.1)	29.2% (12.6–51.1)^a^	8.3% (1.0–27.0)
Geometric mean titer (95% CI)	9.0 (5.3–15.4)	5.3 (4.7–5.9)	5.0 (5.0–5.0)	9.4 (6.8–12.9)	13.4 (9.3–19.3)^a^	6.2 (5.2–7.5)	7.5 (5.5–10.2)	17.8 (10.9–29.2)^a^	6.1 (4.8–7.9)
3 weeks later									
Seroprotection rate % (95% CI)	7.7% (0.2–36.0)	0.0% (0–24.7)	0.0% (0–24.7)	54.0% (39.3–68.2)^a^	58.0% (43.2–71.8)	4.0% (0.5–13.7)	33.3% (15.6–55.3)^a^	58.3% (36.6–77.9)	12.5% (2.7–32.4)
Geometric mean titer (95% CI)	7.7 (4.5–13.0)	5.3 (4.7–5.9)	5.0 (5.0–5.0)	29.5 (18.9–46.0)^a^	37.3 (23.5–59.2)^a^	7.6 (6.2–9.2)	18.3 (10.4–32.4)^a^	43.6 (24.6–77.5)^a^	6.5 (4.8–8.7)
Fold increase of GM titer(95% CI)	0.9 (0.7–1.1)	1.0 (1.0–1.0)	1.0 (1.0–1.0)	3.1 (2.1–4.6)^a^	2.8 (1.8–4.4)^a^	1.2 (1.0v1.5)	2.4 (1.4–4.3)^a^	2.4 (1.5–4.1)^a^	1.1 (0.9–1.3)
Seroresponse rate % (95% CI)	0.0% (0–24.7)	0.0% (0–24.7)	0% (0–24.7)	42.0% (28.2–56.8)^a^	44.0% (30.0–58.8)^a^	12.0% (4.5–24.3)	41.7% (22.1–63.4)^a^	41.7% (22.1–63.4)^a^	4.2% (0.1–21.1)
Seroconversion rate % (95% CI)	0% (0–24.7)	0.0% (0–24.7)	0% (0–24.7)	36.0% (22.9–50.8)^a^	36.0% (22.9–50.8)^a^	0.0% (0.0–7.1)	20.8% (7.1–42.2)	33.3% (15.6–55.3)^a^	4.2% (0.1–21.1)
6 weeks later									
Seroprotection rate % (95% CI)	7.7% (0.2–36.0)	0.0% (0–24.7)	0.0% (0–24.7)	46.0% (31.8–60.7)^a^	58.0% (43.2–71.8)	6.0% (1.3–16.6)	37.5% (18.8–59.4)^a^	58.3% (36.6–77.9)	4.2% (0.1–21.1)
Geometric mean titer (95% CI)	7.7 (4.6–12.6)	6.2 (4.5–8.5)	5.0 (5.0–5.0)	21.4 (14.6–31.4)^a^	40.6 (25.3–65.1)^a^	6.9 (5.7–8.3)	20.0 (10.5–37.9)^a^	43.6 (22.2–85.8)^a^	6.1 (4.9–7.6)
Fold increase of GM titer (95% CI)	0.9 (0.6–1.2)	1.2 (0.9–1.5)	1.0 (1.0–1.0)	2.3 (1.6–3.3)^a^	3.0 (1.9–4.9)^a^	1.1 (0.9–1.3)	2.7 (1.5–4.9)^a^	2.4 (1.4–4.3)^a^	1.0 (0.8–1.3)
Seroresponse rate % (95% CI)	0.0% (0–24.7)	7.7% (0.2–36.0)	0.0% (0–24.7)	38.0% (24.7–52.8)^a^	42.0% (28.2–56.8)^a^	6.0% (1.3–16.6)	37.5% (18.8–59.4)^a^	41.7% (22.1–63.4)	4.2% (0.1–21.1)
Seroconversion rate % (95% CI)	0.0% (0–24.7)	0.0% (0–24.7)	0.0% (0–24.7)	30.0% (17.9–44.6)^a^	38.0% (24.7–52.8)^a^	0.0% (0.0–7.1)	25.0% (9.8–46.7)	37.5%(18.8–59.4)^a^	0.0% (0.0–14.3)
9 weeks later									
Seroprotection rate % (95% CI)	7.7% (0.2–36.0)	0.0% (0.0–24.7)	0.0% (0.0–24.7)	46.0% (31.8–60.7)^a^	62.0% (47.2–75.4)	10.0% (3.3–21.8)	37.5% (18.8–59.4)^a^	66.7% (44.7–84.4)	16.7% (4.7–37.4)
Geometric mean titer (95% CI)	9.0 (4.9–16.6)	5.3 (4.7–5.9)	5.0 (5.0–5.0)	21.7 (14.6–32.5)^a^	41.1 (25.8–65.4)^a^	7.4 (5.9–9.3)^a^	20.6 (10.7–39.6)^a^	50.4 (27.4–92.6)^a^	8.4 (5.6–12.6)
Fold increase of GM titer (95% CI)	1.0 (0.8–1.2)	1.0 (1.0v1.0)	1.0 (1.0–1.0)	2.3 (1.6–3.4)^a^	3.1 (1.9–5.0)^a^	1.2 (1.0–1.4)	2.7 (1.5–5.1)^a^	2.8 (1.7–4.6)^a^	1.4 (0.9–2.1)
Seroresponse rate % (95% CI)	0.0% (0.0–24.7)	0.0% (0.0–24.7)	0.0% (0.0–24.7)	40.0% (26.4–54.8)^a^	36.0% (22.9–50.8)^a^	8.0% (2.2–19.2)	37.5% (18.8–59.4)^a^	50.0% (29.1–70.9)^a^	16.7% (4.7–37.4)
Seroconversion rate % (95% CI)	0.0% (0.0–24.7)	0.0% (0.0–24.7)	0.0% (0.0–24.7)	32.0% (19.5–46.7)^a^	34.0% (21.2–48.8)^a^	2.0% (0.1–10.7)	29.2% (12.6–51.1)^a^	45.8% (25.6–67.2)^a^	0.0% (0.0–14.3)
18 weeks later									
Seroprotection rate % (95% CI)	7.7% (0.2–36.0)	0.0% (0.0–24.7)	7.7% (0.2–36.0)	16.0% (7.2–29.1)	30.0% (17.9–44.6)	4.0% (0.5–13.7)	20.8% (7.1–41.2)	33.3% (15.6–55.3)	0.0% (0.0–14.3)
Geometric mean titer (95% CI)	8.1 (4.8–13.6)	5.0 (5.0-5.0)	6.2 (3.9–9.9)	9.3 (6.5–13.4)	11.7 (8.2–16.5)	5.5 (4.8–6.3)	13.4 (7.7–23.2)^a^	18.9 (9.7–36.6)	5.0 (5.0–5.0)
Fold increase of GM titer (95% CI)	0.9 (0.8–1.1)	0.9 (0.8–1.1)	1.2 (0.8–2.0)	1.0 (0.7–1.5)	0.9 (0.5–1.4)	0.9 (0.8–1.0)	1.8 (1.0–3.2)^a^	1.1 (0.6–1.9)	0.8 (0.6–1.1)
Seroresponse rate % (95% CI)	0.0% (0.0–24.7)	0.0% (0.0–24.7)	7.7% (0.2–36.0)	14.0% (5.8–26.7)	16.0% (7.2–29.1)	0.0% (0.0–7.1)	29.2% (12.6–51.1)^a^	20.8% (7.1–42.2)	0.0% (0.0–14.3)
Seroconversion rate % (95% CI)	0.0% (0.0–24.7)	0.0% (0.0–24.7)	0.0% (0.0–24.7)	10.0% (3.3–21.8)	12.0% (4.5–24.3)	0.0% (0.0–7.1)	16.7% (4.7–37.4)	16.7% (4.7–37.4)	0.0% (0.0–14.3)

Abbreviations: CI: confidence interval; GM: geographic mean; Definition of seroprotection: HI titers ≥ 1:40; Seroresponse: ≥ 4-fold increase in antibody titer after vaccination; Seroconversion: ≥ 4-fold or more increase in HI titer and HI titer ≥ 1:40 after vaccination.

^a^P value < 0.05 compared with the unvaccinated group.

^b^There were no statistical differences between all immunogenic profiles between the one-dose and two-dose vaccination groups.

**Table 4:** Comparison of hemagglutination-inhibition (HI) immunogenicity in normal individuals and dialysis patients three weeks after receiving one dose of trivalent seasonal influenza vaccine.

Immunogenicity end point	Normal adult population (n = 66)			Dialysis adult population (n = 76)		
	H1N1	H3N2	B	H1N1	H3N2	B
Baseline						
Seroprotection rate % (95% CI)	16.7% (8.6–27.9)	31.8% (20.9–44.4)	13.6% (6.4–24.3)	22.4% (13.6–33.4)	30.3% (20.3–41.9)	9.2% (3.8–18.1)
Geometric mean titer (95% CI)	10.8 (8.1–14.4)	16.5 (11.7–23.3)	8.4 (6.7–10.4)	12.3 (9.6–15.8)	15.2 (11.3–20.5)	7.7 (6.4–9.4)
3 weeks later						
Seroprotection rate % (95% CI)	92.4% (83.2–97.5)	84.9% (73.9–92.5)	42.4% (30.3–55.2)	67.1% (55.4–77.5)^a^	68.4% (56.8–78.6)	23.7% (14.7–34.8)
Geometric mean titer (95% CI)	155.0 (115.5–208.0)	152.1 (106.4–217.4)	26.8 (20.4–35.3)	48.4 (35.2–66.7)^a^	51.6 (36.5–73.2)^a^	11.8 (9.0–15.4)^a^
Fold increase of GM titer (95% CI)	14.3 (9.7–21.1)	8.9 (5.9–13.5)	3.2 (2.4–4.4)	3.9 (2.8–5.5)^a^	3.4 (2.4–4.9)^a^	1.5 (1.2–2.0)^a^
Seroresponse rate % (95% CI)	78.8% (67.0–87.9)	72.7% (60.4–83.0)	47.0% (34.6–59.7)	50.0% (38.3–61.7)^a^	39.5% (28.4–51.4)^a^	23.7% (14.7–34.8)^a^
Seroconversion rate % (95% CI)	77.3% (65.3–86.7)	65.2% (52.4–76.5)	28.8% (18.3–41.2)	42.1% (30.9–54.0)^a^	34.2% (23.7–46.0)^a^	15.8% (8.4–26.0)
	**Normal elderly population (n = 48)**			**Dialysis elderly population (n = 74)**		
**Immunogenicity end point**	**H1N1**	**H3N2**	**B**	**H1N1**	**H3N2**	**B**
Baseline						
Seroprotection rate % (95% CI)	25.0% (13.6–39.6)	56.3% (41.2–70.5)	14.6% (6.1–27.8)	12.2% (5.7–21.8)	31.1% (20.8–42.9)^a^	8.1% (3.0–16.8)
Geometric mean titer (95% CI)	13.4 (9.7–18.4)	26.9 (18.4–39.2)	9.6 (6.9–13.3)	8.7 (6.9–11.0)^a^	14.7 (11.0–19.6)^a^	6.2 (5.4–7.2)^a^
3 weeks later						
Seroprotection rate % (95% CI)	81.3% (67.4–91.1)	87.5% (74.8–95.3)	29.2% (17.0–44.1)	47.3% (35.6–59.3)	58.1% (46.1–69.5)	6.8% (2.2–15.1)^a^
Geometric mean titer (95% CI)	92.4 (64.3–132.9)	108.8 (78.2–151.6)	18.5 (13.2–26.0)	25.3 (17.8–35.8)^a^	39.3 (27.5–56.1)	7.2 (6.1–8.5)^a^
Fold increase of GM titer (95% CI)	6.9 (4.6–10.3)	4.0 (2.7–6.0)	1.9 (1.4–2.6)	2.9 (2.1–4.0)^a^	2.7 (1.9–3.8)	1.2 (1.0–1.3)^a^
Seroresponse rate % (95%CI)	60.4% (45.3–74.2)	47.9% (33.3–62.8)	18.8% (9.0–32.6)	41.9% (30.5–53.9)^a^	43.2% (31.8–55.3)	9.5% (3.9–18.5)
Seroconversion rate % (95%CI)	60.4% (45.3-74.2)	39.6% (25.8–54.7)	14.6% (6.1–27.8)	31.1% (20.8–42.9)^a^	35.1% (24.4–47.1)	1.4% (0.0–7.3)^a^

Abbreviations: CI: confidence interval; GM: geographic mean; Definition of seroprotection: HI titers ≥ 1:40; Seroresponse: ≥ 4-fold increase in antibody titer after vaccination; Seroconversion: ≥ 4-fold or more increase in HI titer and HI titer ≥ 1:40 after vaccination.

^a^P value < 0.05 when compared with the normal population.

**Table 5:** Determinants of seroprotection and seroresponse by the multivariate logistic regression models with generalized estimating equations in hemodialysis patients receiving either one (n = 86) or two doses (n = 64) of influenza vaccination.

Variable	H1N1		H3N2		B	
	Seroprotection	Seroresponse	Seroprotection	Seroresponse	Seroprotection	Seroresponse
	OR (95% CI)	OR (95% CI)	OR (95% CI)	OR (95% CI)	OR (95% CI)	OR (95% CI)
Vaccination schedule (2 vs. 1 dose)	1.01 (0.58–1.75)	1.14 (0.65–2.00)	1.30 (0.78–2.17)	1.34 (0.76–2.36)	1.33 (0.58–3.07)	1.36 (0.65–2.82)
Age (year)	0.98 (0.95–1.00)	0.97 (0.95–1.00)	0.99 (0.97–1.01)	0.99 (0.97-1.02)	0.95 (0.92–0.97)	0.96 (0.93–0.98)
Seroprotection before vaccination	5.31 (3.29–8.57)	0.13 (0.04–0.38)	4.57 (2.84–7.37)	0.18 (0.09–0.36)	25.25 (8.01–79.57)	0.13 (0.01–1.17)
Total–cholesterol (mg/dL)	1.00 (0.99–1.01)	1.00 (1.00–1.01)	1.01 (1.00–1.01)	1.00 (1.00–1.01)	1.00 (0.99–1.02)	1.01 (1.00–1.02)
Hematocrit (%)	0.98 (0.93–1.03)	0.96 (0.90–1.01)	1.02 (0.97–1.07)	0.97 (0.91–1.04)	1.01 (0.93–1.10)	1.00 (0.90–1.07)
Ferritin (g/dl)	0.98 (0.94–1.05)	1.03 (0.96–1.10)	1.01 (0.95–1.07)	1.03 (0.97–1.10)	0.94 (0.85–1.03)	0.96 (0.86–1.08)

Abbreviation: OR: Odds ratio; Definition of seroprotection: hemagglutination-inhibition titers ≥ 1:40; seroresponse: ≥ 4-fold increase in antibody titer after vaccination.

**Table 6:** Systemic and local adverse effects within one week after influenza vaccination in the dialysis patients.

	The first dose at week 0 (n = 170)		The second dose at 3 weeks later (n = 70)	
	Mild^a^	Moderate to severe^a^	Mild^a^	Moderate to severe^a^
Systemic adverse effect				
Fever (> 38 °C)	1.80%	0.00%	0.00%	0.00%
Nasal congestion	4.10%	1.80%	2.90%	0.00%
Cough	6.50%	1.20%	5.70%	0.00%
Sore throat	4.10%	1.20%	2.90%	0.00%
Muscle aches	11.80%	2.40%	11.40%	0.00%
Headache	2.90%	2.40%	1.40%	1.40%
Nausea	1.80%	1.20%	1.40%	1.40%
Vomiting	2.90%	1.20%	1.40%	1.40%
Malaise	17.70%	1.80%	12.90%	0.00%
Eye redness	1.20%	0.00%	0.00%	0.00%
Chest tightness	1.80%	1.20%	1.40%	0.00%
Respiratory distress	1.80%	1.20%	1.40%	0.00%
Face edema	0.00%	0.00%	0.00%	0.00%
Local adverse effect				
Pain	17.70%	0.00%	18.60%	0.00%
Swelling	2.90%	0.00%	2.90%	0.00%
Redness	0.00%	0.00%	0.00%	0.00%
Ecchymosis	0.60%	0.00%	1.40%	0.00%
Decrease limb mobility	1.20%	0.00%	1.40%	0.00%

^a^The severity of the symptoms were defined as: (a) mild: symptoms are easily tolerable; (b) moderate: symptoms interfere with the daily activity; (c) severe: unable to work or perform daily activity due to symptoms. In the item of “fever”, ≥ 38.0 °C ~ 39.0 °C was defined as mild, >39.0 °C ~ 40.0 °C as moderate and > 40.0 °C as seve
