# Supplementary material for: Changes of immunogenic profiles between a single dose and one booster influenza vaccination in hemodialysis patients – an 18-week, open-label trial
Source: Sci Rep. 2016 Feb 12;6:20725. doi: 10.1038/srep20725 (PMC4751607; doi:10.1038/srep20725)
Supplement: Supplementary Information [file srep20725-s1.doc]

**The title of the manuscript :**

Changes of immunogenic profiles between a single dose and one booster influenza vaccination in hemodialysis patients – an 18-week, open-label trial.

**The author list:**

Yu-Tzu Chang, MD, MSc^1,2^, Jen-Ren Wang, PhD^3,4^, Meng-Te Lin, MD^5^, Chi-Jung Wu, MD, PhD^2,4^, Ming-Song Tsai, MD^5^, Wen-Chi Chiang Lin, MD^5^, Te-En Shih, MD^5^, Te-Hui Kuo, MD, MSc^2^, Eing-Ju Song, PhD^6^ and Junne-Ming Sung, MD^2,5^

**Supplementary Table 1.** Comparison of secular changes of log_10_-transformed hemagglutination-inhibition antibody titers between different time points by multivariate generalized estimating equations for linear regression in patients receiving different dosages of influenza vaccination^a^.

|  | **H1N1** |  | **H3N2** |  | **B** |  |
| --- | --- | --- | --- | --- | --- | --- |
| **Variable** | β | *P value* | β | *P value* | β | *P value* |
| The unvaccinated group |  |  |  |  |  |  |
| Week 0 | 0 | - | 0 | - | 0 | - |
| Week 3^b^ | -0.0878 | 0.0518 | -0.0125 | 0.7812 | 0.0125 | 0.307 |
| Week 6^b^ | -0.0753 | 0.0396 | 0.0376 | 0.5089 | 0.0000 | 1.000 |
| Week 9^b^ | -0.0125 | 0.6534 | 0.0125 | 0.6534 | 0.0000 | 1.000 |
| Week 18^b^ | -0.0753 | 0.0897 | -0.0376 | 0.4328 | 0.0125 | 0.307 |
| The one-dose vaccination group |  |  |  |  |  |  |
| Week 0^b^ | 0 | - | 0 | - | 0 | - |
| Week 3^b^ | 0.5543 | <.0001 | 0.4910 | <.0001 | 0.1183 | 0.0015 |
| Week 6^b^ | 0.4289 | <.0001 | 0.5196 | <.0001 | 0.0968 | 0.0102 |
| Week 9^b^ | 0.4146 | <.0001 | 0.4945 | <.0001 | 0.1218 | 0.0005 |
| Week 18^b^ | 0.0168 | 0.8317 | -0.0753 | 0.3242 | -0.0609 | 0.0226 |
| The two-dose vaccination group |  |  |  |  |  |  |
| Week 0^b^ | 0 | - | 0 | - | 0 | - |
| Week 3^b^ | 0.5033 | <.0001 | 0.4751 | <.0001 | 0.1364 | 0.0218 |
| Week 6^b^ | 0.4845 | <.0001 | 0.5597 | <.0001 | 0.1411 | 0.0066 |
| Week 9^b^ | 0.5456 | <.0001 | 0.5550 | <.0001 | 0.1552 | 0.0190 |
| Week 18^b^ | 0.1035 | 0.2594 | 0.0094 | 0.9217 | -0.0800 | 0.1486 |

^a^ All models were adjusted for age, seroprotection before vaccination, total cholesterol, hematocrit and ferritin.

^b^ β coefficient values and theirs corresponding *p* values at different time points were estimated when comparing with the baseline period (week 0).
